# Exactly solvable statistical physics models for large neuronal populations

**DOI:** 10.1103/PhysRevResearch.7.L022039

**Published:** 2025-05-19

**Authors:** Christopher W. Lynn, Qiwei Yu, Rich Pang, William Bialek, Stephanie E. Palmer

**Affiliations:** 1 Department of Physics, Quantitative Biology Institute, and Wu Tsai Institute, Yale University, New Haven, Connecticut 06520, USA; 2 Initiative for the Theoretical Sciences, The Graduate Center, City University of New York, New York, New York 10016, USA; 3 Joseph Henry Laboratories of Physics, Princeton University, Princeton, New Jersey 08544, USA; 4 Lewis-Sigler Institute for Integrative Genomics, Princeton University, Princeton, New Jersey 08544, USA; 5 Princeton Neuroscience Institute, Princeton University, Princeton, New Jersey 08544, USA; 6 Center for Studies in Physics and Biology, Rockefeller University, New York, New York 10065, USA; 7 Department of Organismal Biology and Anatomy, University of Chicago, Chicago, Illinois 60637, USA; 8 Department of Physics, University of Chicago, Chicago, Illinois 60637, USA

## Abstract

Maximum-entropy methods provide a principled path connecting measurements of neural activity directly to statistical physics models, and this approach has been successful for populations of N~100 neurons. As N increases in new experiments, we enter an undersampled regime where we have to choose which observables should be constrained in the maximum-entropy construction. The best choice is the one that provides the greatest reduction in entropy, defining a “minimax entropy” principle. This principle becomes tractable if we restrict attention to correlations among pairs of neurons that link together into a tree; we can find the best tree efficiently, and the underlying statistical physics models are exactly solved. We use this approach to analyze experiments on N~1500 neurons in the mouse hippocampus, and we find that the resulting model captures key features of collective activity in the network.

It has long been hoped that neural networks in the brain could be described using concepts from statistical physics [1–7]. More recently, our ability to explore the brain has been revolutionized by experimental techniques that record the activity from thousands of individual neurons, simultaneously [8–14]. Maximum-entropy methods connect these data to theory, starting with measured properties of the network and arriving at models that are mathematically equivalent to statistical physics problems [15,16]. In some cases, these models provide successful, parameter-free predictions for many detailed features of the neural activity patterns [7,17,18]. The same ideas have been used in contexts ranging from the evolution of protein families to ordering in flocks of birds [19–25]. But as experiments probe systems with more and more degrees of freedom, the number of samples that we can collect typically does not increase in proportion. Here we present a strategy for building maximum entropy models in this undersampled regime, and we apply this strategy to data from 1000+ neurons in the mouse hippocampus.

Consider a system of N variables $x=\left\{x_{i}\right\},i=1,2,\ldots ,N$. To describe the system, we would like to write down the probability distribution P(x) over these microscopic degrees of freedom, in the same way that we write the Boltzmann distribution for a system in equilibrium. In the example that we discuss below, all the $x_{i}$ are observed at the same moment in time, but we could also include time in the index i, so that P(x) becomes a probability distribution of trajectories.

From these N variables, we can construct a set of K operators or observables $\left\{f_{v}(x)\right\},v=1,2,\ldots ,K$. The maximum-entropy approach takes this limited number of observables seriously, and insists that expectation values for these quantities predicted by the model match the values measured in experiment,

(1) $${\left\langle f_{v}(x)\right\rangle }_{P}={\left\langle f_{v}(x)\right\rangle }_{\text{exp}},$$

or more explicitly,

(2) $$\sum_{x}P(x)f_{v}(x)=\frac{1}{M}\sum_{m=1}^{M}f_{v}\left(x^{\left(m\right)}\right),$$

where $x^{\left(m\right)}$ is the mth sample out of M samples in total. Notice that to have control over errors in the measurement of all K expectation values, we need to have K≪MN.

There are infinitely many distributions that obey these matching conditions, but the idea of maximum entropy is that we should choose the one that has the least possible structure, or equivalently generates samples that are as random as possible while obeying the constraints in Eq. (1). From Shannon we know that “as random as possible” translates uniquely to finding the distribution that has the maximum entropy consistent with the constraints [26,27]. The solution of this optimization problem has the form

(3) $$P(x)=\frac{1}{Z}\text{exp}\left[-\sum_{v=1}^{K}\lambda_{v}f_{v}(x)\right],$$

where the coupling constants $\lambda_{\nu }$ must be chosen to satisfy Eq. (1). We emphasize that in using this approach, the model in Eq. (3) is something that needs to be tested—in systems such as networks of neurons, there is no H-theorem telling us that entropy will be maximized, nor is there a unique choice for the constraints that would correspond to the Hamiltonian of an equilibrium system.

Without a Hamiltonian, how should we choose the observables $f_{\nu }$? Probability distributions define a code for the data [26] in which each state x is mapped to a code word of length

(4) ℓ(x)=-logP(x),

so the mean code length for the data is

(5) $$\langle \ell \rangle_{\text{exp}}\equiv -\frac{1}{M}\sum_{n=1}^{M}\text{log}\;P\left(x^{\left(n\right)}\right).$$

Combining Eqs. (1) and (3), $\langle \ell \rangle_{\text{exp}}$ is exactly the entropy of P. Thus, among all maximum entropy distributions, the one that gives the shortest description of the data is the one with minimum entropy. This “minimax entropy” principle was discussed 25 years ago [28], but has attracted relatively little attention.

Every time we add a constraint, the maximum possible entropy is reduced [29], and this entropy reduction is an information gain. Thus the minimax entropy principle tells us to choose observables whose expectation values provide as much information as possible about the microscopic variables. The problem is that finding these maximally informative observables is generally intractable: One must search over all possible sets of observables, and for each set one must solve the maximum-entropy problem for the parameters $\lambda_{\nu }$.

To make progress, we restrict the class of observables that we consider. With populations of N~100 neurons, it can be very effective to constrain just the mean activity of each neuron and the correlations among pairs [16–18]. But this corresponds to $K\propto N^{2}$ constraints, and at large N we will violate the good sampling condition K≪NM. To restore good sampling, we try constraining only $N_{c}$ of the correlations, which define links between specific pairs of neurons that together form a graph 𝒢. If we describe the individual neurons as being either active or silent, so that $x_{i}\in \{0,1\}$, then the maximum entropy distribution is an Ising model on the graph 𝒢,

(6) $$P_{𝒢}(x)=\frac{1}{Z}\text{exp}\left[\sum_{(ij)\in 𝒢}J_{\text{ij}}x_{i}x_{j}+\sum_{i}h_{i}x_{i}\right],$$

where $\left\{h_{i},J_{\text{ij}}\right\}$ must be adjusted to match the measured expectation values ${\left\langle x_{i}\right\rangle }_{\text{exp}}$ and ${\left\langle x_{i}x_{j}\right\rangle }_{\text{exp}}$ for pairs (ij)∈𝒢. The minimax entropy principle tells us that we should find the graph 𝒢 with a fixed number of links $N_{c}$ such that $P_{𝒢}(x)$ has the smallest entropy while obeying these constraints. Even this problem remains intractable.

Calculations in statistical physics are hard due to feedback loops. Without loops, one can often make exact and efficient calculations, as in one-dimensional systems or on Bethe lattices [30]. If the graph 𝒢 has no loops, then it describes a tree 𝒯, and we can analytically solve the maximum-entropy problem for the fields $h_{i}$ and couplings $J_{\text{ij}}$ as functions of the observables $\left\langle x_{i}\right\rangle$ and $\left\langle x_{i}x_{j}\right\rangle$,

(7) $$J_{\text{ij}}=\text{ln}\left[\frac{\left\langle x_{i}x_{j}\right\rangle \left(1-\left\langle x_{i}\right\rangle -\left\langle x_{j}\right\rangle +\left\langle x_{i}x_{j}\right\rangle \right)}{\left(\left\langle x_{i}\right\rangle -\left\langle x_{i}x_{j}\right\rangle \right)\left(\left\langle x_{j}\right\rangle -\left\langle x_{i}x_{j}\right\rangle \right)}\right],$$

(8) $$h_{i}=\text{ln}\frac{\left\langle x_{i}\right\rangle }{1-\left\langle x_{i}\right\rangle }+\sum_{j\in {𝒩}_{i}}\text{ln}\left[\frac{\left(1-\left\langle x_{i}\right\rangle \right)\left(\left\langle x_{i}\right\rangle -\left\langle x_{i}x_{j}\right\rangle \right)}{\left\langle x_{i}\right\rangle \left(1-\left\langle x_{i}\right\rangle -\left\langle x_{j}\right\rangle +\left\langle x_{i}x_{j}\right\rangle \right)}\right],$$

where ${𝒩}_{i}$ are the neighbors of i on the tree [15,31]. Moreover, we can write the entropy of the model $P_{𝒯}$ as

(9) $$S_{𝒯}=-\sum_{x}P_{𝒯}(x)\text{log}\;P_{𝒯}(x)=S_{\text{ind}}-\sum_{(ij)\in 𝒯}I_{\text{ij}},$$

where $S_{\text{ind}}$ is the independent entropy of the individual variables, and $I_{\text{ij}}$ is the mutual information between $x_{i}$ and $x_{j}$ [Fig. 1(a)]. Due to the maximum-entropy constraints, the mutual information $I_{\text{ij}}$ in the model matches that in the data for (ij)∈𝒯. Thus, we can compute the entropy $S_{𝒯}$ of any maximum-entropy model on a tree without constructing the model itself.

To solve the minimax entropy problem, we want to find the tree 𝒯 that minimizes the entropy $S_{𝒯}$ in Eq. (9), or, equivalently, maximizes the total mutual information

(10) $$I_{𝒯}=\sum_{(ij)\in 𝒯}I_{\text{ij}}.$$

We have therefore simplified the minimax entropy problem to a minimum spanning tree problem [15], which admits a number of efficient solutions. For example, rather than searching over all $N^{N-2}$ possible trees, one can use Prim’s algorithm (Fig. 1) to find the tree 𝒯 with maximum information $I_{𝒯}$ in $O\left(N^{2}\right)$ time [32]. This means that if the data are generated by an Ising model on a tree, then we are guaranteed to find the true interactions and our model is exact [33]. Thus, by restricting observables to pairwise correlations that form a tree, we can exactly solve not only the maximum-entropy problem [using Eqs. (7) and (8)], but also the minimax entropy problem for the optimal tree 𝒯, even at very large N.

As emphasized above, our approach yields a model that needs to be tested. While maximum-entropy models traditionally constrain all N(N-1)/2 pairwise correlations $\left\langle x_{i}x_{j}\right\rangle$ [16–18], here we only constrain the N-1 corresponding to (ij)∈𝒯. At large N, this sparsity guarantees that we are well-sampled, but it also means that we only have access to a vanishingly small fraction 2/N of all pairwise correlations. Can such a sparse backbone of correlations contain enough information to capture features of the collective activity in the population?

To answer this question, we analyze calcium imaging data from CA1 in the mouse hippocampus as the animal runs along a virtual track [34]. Discussions of the hippocampus often focus on place cells, yet only about 30% of pyramidal cells in CA1 exhibit significant place fields in a single environment [35–38]. While individual place cells can be viewed as responding independently to external cues, to understand the collective activity of a mixed population one must consider interactions between neurons. This approach has been widely employed to study collective hippocampal functions including memory storage, pattern separation, and pattern completion [39–41]. Moreover, previous applications of the maximum-entropy principle in populations of N~100 neurons have shown that the detailed patterns of activity can sometimes be explained entirely by the pairwise correlations between neurons [17,18].

Here, we consider calcium imaging from a population of N=1485 neurons with images collected at 30 Hz for 39 min, yielding $M\approx 7\times 10^{4}$ samples. The signal from each cell naturally binarizes into active/silent $\left(x_{i}=1/0\right)$ in each video frame [17], and we have sufficient data to estimate the mutual information $I_{\text{ij}}$ with small errors [33]. Among all $\sim 10^{6}$ pairs of neurons, only 9% exhibit significant mutual information $I_{\text{ij}}$ [Fig. 2(a); see [33] for details]. We see that the distribution of mutual information is heavy-tailed, which tells us that a small number of correlations contain orders of magnitude more information than average ($\overset{‾}{I}=2.9\times 10^{-4}$ bits). Additionally, while most pairs of neurons are negatively correlated [Fig. 2(b)], the strongest values of $I_{\text{ij}}$ belong to pairs that are positively correlated [Fig. 2(c)], possibly reflecting the excitatory nature of pyramidal cells. Together, these observations suggest that a sparse network of positively correlated neurons may provide a large amount of information about the collective neural activity.

Applying our method, we identify the tree of maximally informative correlations [Fig. 3(a)], which captures $S_{\text{ind}}-S_{𝒯}=I_{𝒯}=26.2$ bits of information; this is more than 50 times the average we find on a random tree [Fig. 3(b)]. Despite including only 2/N≈0.1% of all pairwise correlations, we find that the optimal tree reduces the entropy of independent neurons by $I_{𝒯}/S_{\text{ind}}\approx 14.4\%$; this reduction in entropy is as strong as freezing the states of 214 randomly selected neurons.

In the model of Eq. (6), positive (negative) $J_{\text{ij}}$ means that activity in neuron i leads to activity (silence) in neuron j. For random trees, the interactions $J_{\text{ij}}$ are split almost evenly between positive and negative [Fig. 3(b)]; this is consistent with previous investigations of fully connected modes in smaller systems of N~100 neurons [16,18,42]. But since the largest mutual information is associated with positive correlations [Fig. 2(c)], the optimal tree produces a model with strong interactions that are almost exclusively positive [Fig. 3(a)]. We have arrived at an Ising ferromagnet, with excitatory interactions similar to those between pyramidal neurons.

While the model $P_{𝒯}$ is defined to match a sparse subset of the observed correlations for (ij)∈𝒯, we can ask what $P_{𝒯}$ predicts for all pairs of neurons. The optimal tree underestimates the strength of weak correlations, yet accurately predicts the strong correlations in the population [Fig. 3(c)]. Meanwhile, a typical set of correlations (that is, a random tree) captures almost none of the overall correlation structure in the data [Fig. 3(c)].

At the level of individual neurons, the model makes a clear prediction for the probability that a neuron spikes in response to the activity of the rest of the network:

(11) $$P_{𝒯}\left(x_{i}=1|x_{-i}\right)={\left[1+\text{exp}\left(-h_{i}^{\text{eff}}\left(x_{-i}\right)\right)\right]}^{-1},$$

where $x_{-i}$ represents the states of all neurons other than i, and $h_{i}^{\text{eff}}\left(x_{-i}\right)=h_{i}+\sum_{j\in {𝒩}_{i}}J_{\text{ij}}x_{j}$ is an effective field. In a tree, each neuron only interacts with two other neurons in the population (on average). Although this sparsity restricts the range of predictions for random trees, the optimal tree still exhibits good agreement between theory and data across a wide range of spike probabilities [Fig. 3(d)]. These results suggest that a large amount of information about the neural activity is packed into a sparse network of strong correlations.

To interpret these results, we apply our framework to a canonical model of complex interacting systems: the Sherrington-Kirkpatrick (SK) model, which is equivalent to an Ising system with all-to-all interactions $J_{\text{ij}}$ drawn from a zero-mean Gaussian [43]. We can investigate the impact of sparsity in the network by randomly removing interactions, resulting in a connection density d [Fig. 4(a)]. Simulating the original SK model (d=1) with N=1485 artificial neurons and $M\approx 7\times 10^{4}$ samples to match the neural data, we find that the optimal tree captures $I_{𝒯}/S_{\text{ind}}\approx 4.2\%$ of the independent entropy [Fig. 4(b)]; by contrast, a random tree only captures 0.5%. As we remove interactions from the system (decreasing d), the optimal tree uncovers a larger proportion of the interactions, thereby capturing more information about correlations in the system [Fig. 4(b)]; meanwhile, random trees becomes even less effective.

For the optimal tree to achieve the fraction $I_{𝒯}/S_{\text{ind}}\approx 14.4\%$ observed in data [Fig. 3(a)], one must remove 99% of the interactions from the SK model (d=0.01). For N=1485, this corresponds to each neuron interacting with an average of only 15 other cells in the population. At d=0.01, while the optimal tree fails to predict weak correlations, it captures both the strong positive and negative correlations in the system [Fig. 4(c)]. Moreover, it provides near-perfect predictions for the spiking probabilities of individual artificial neurons [Fig. 4(d)]. We therefore find that our minimax entropy framework becomes more accurate—resembling the neural data in Fig. 3—for sparse networks of interactions.

Thus far, we have focused on features of individual neurons and neuron pairs, but can our approach predict something about the activity of the population as a whole? One key measure of collective neural activity is the probability P(k) that k out of the N neurons are simultaneously active within a window of time [16,18,25,42]. In the data, we see moments of both synchrony (large k) and silence (small k) that are much more likely than expected from independent neurons (Fig. 5). The optimal tree captures most of this collective behavior, correctly predicting ~100-fold increases in the probability that k≳50 neurons are active at once (Fig. 5, red). By contrast, a random selection of correlations yields predictions that are indistinguishable from independent neurons (Fig. 5, cyan). While the detailed patterns of neural activity are likely shaped by a web of weaker correlations, these results show that a sparse backbone of the strongest correlations can still produce the large-scale synchrony observed in neural systems.

In summary, it has been appreciated for nearly two decades that the maximum-entropy principle provides a link from data directly to statistical physics models, and this is useful in networks of neurons as well as other complex systems [15–25]. Less widely emphasized is that we do not have “the” maximum-entropy model, but rather a collection of possible models depending on what features of the system we choose to constrain. Quite generally, we should choose the features that are most informative, leading to the minimax entropy principle [28]. As we study larger and larger populations of neurons, we enter an undersampled regime in which selecting a limited number of maximally informative features is not only conceptually appealing but also a practical necessity.

The problem is that the minimax entropy principle is intractable in general. Here we have made progress in two steps. First, following previous successes, we focus on constraining the mean activity and pairwise correlations. Second, we take the lesson of the Bethe lattice and select only pairs that define a tree. Once we do this, the relevant statistical mechanics problem is solved exactly, and the optimal tree can be found in quadratic time [15,31]. This means that there is a family of strongly interacting statistical physics models for thousands of neurons that we can construct in seconds on a personal computer. It is worth asking whether these models can capture any of the essential collective behavior in real networks. While our approach has important limitations (see an extended discussion in [33]), we find that the optimal tree correctly predicts strong correlations, individual spike probabilities, and the distribution of synchronous activity (Figs. 3 and 5). The key to this success is the heavy-tailed distribution of mutual information [Fig. 2(a)], and this in turn may be grounded in sparse networks of strong physical connections [44]. While these models cannot capture all aspects of collective behavior, our approach provides at least a starting point for scalable models of the much larger systems now becoming accessible to experiments.

## Figures and Tables

**FIG. 1.**
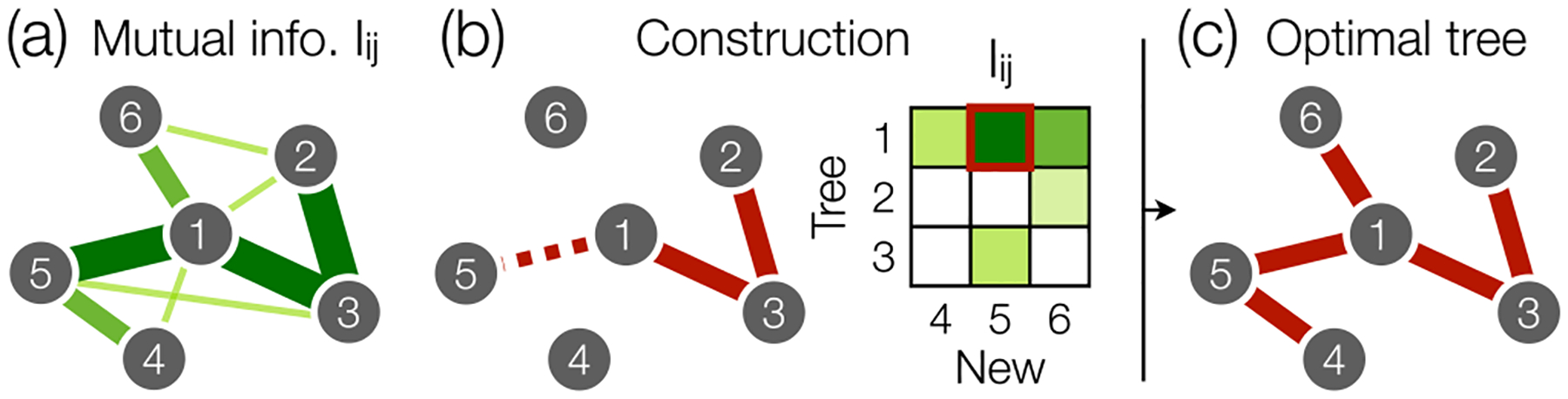
Computing the optimal tree of pairwise correlations. (a) Mutual information between variables in a hypothetical system. (b) One can build the optimal tree one variable at a time, from an arbitrary starting variable, by iteratively adding the connection corresponding to the largest mutual information (dashed) from the tree (solid) to the remaining variables; this is Prim’s algorithm. (c) Optimal tree that minimizes the entropy $S_{𝒯}$ and maximizes the information $I_{𝒯}$.

**FIG. 2.**
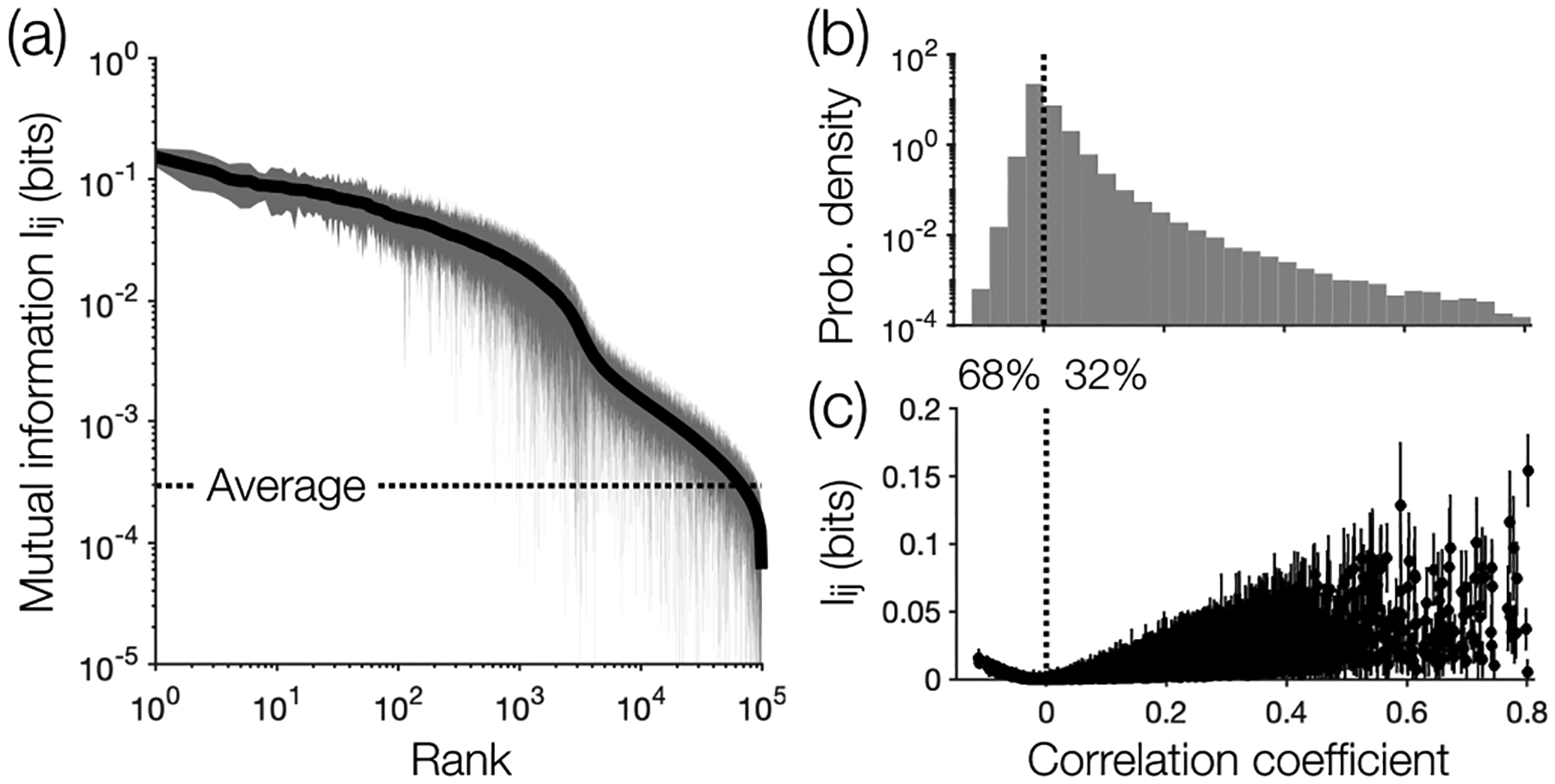
Mutual information in a large population of neurons. (a) Ranked order of the significant mutual information values $I_{\text{ij}}$ in a population of N=1485 neurons in the mouse hippocampus [34]. Solid line and shaded region indicate estimates and errors (two standard deviations) of $I_{\text{ij}}$. (b) Distribution of correlation coefficients over neuron pairs, with percentages indicating the proportion of positively and negatively correlated pairs. (c) Mutual information $I_{\text{ij}}$ vs correlation coefficient, where each point represents a distinct neuron pair. Estimates and errors are the same as in (a).

**FIG. 3.**
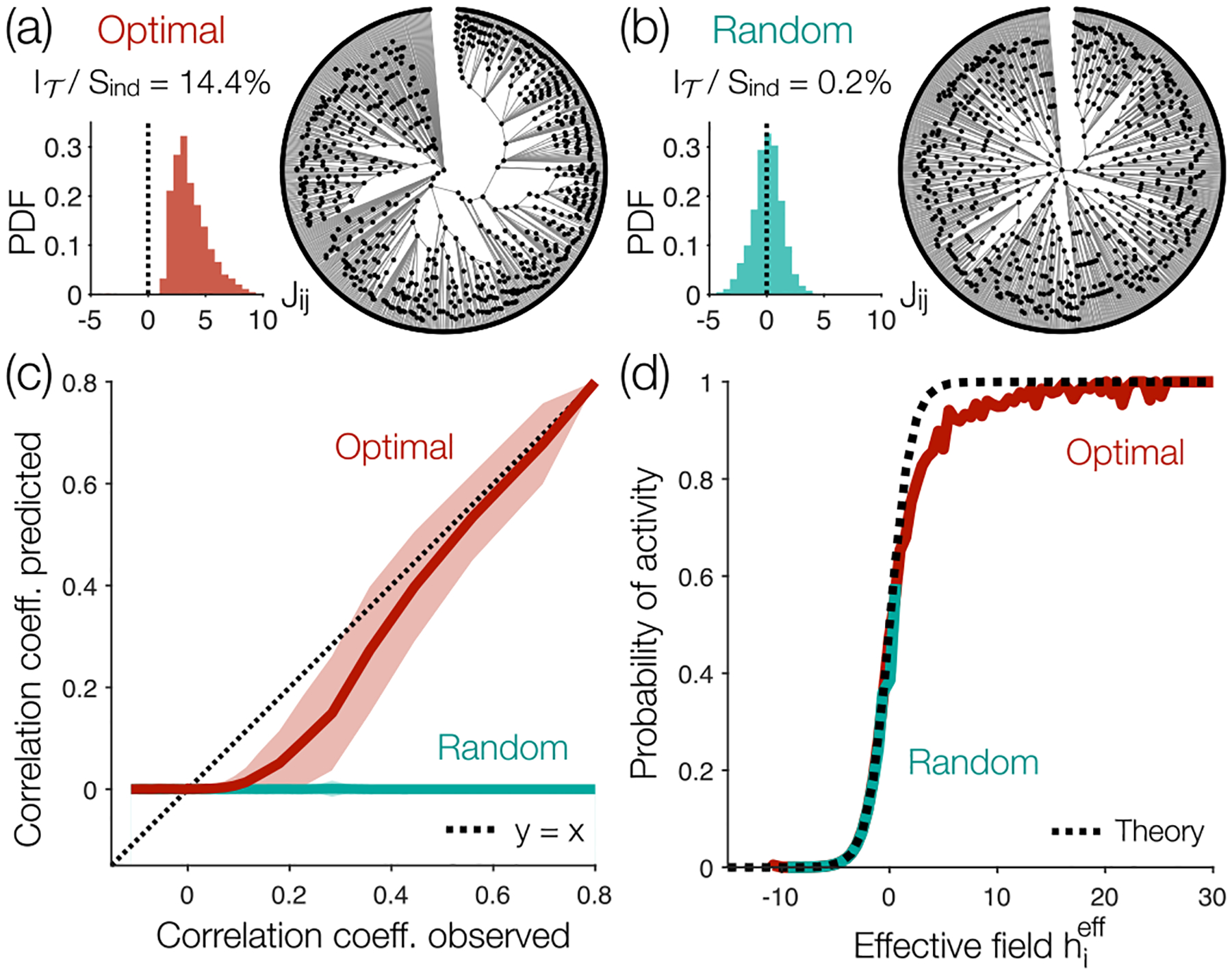
Minimax entropy model of a large neuronal population. (a) Optimal tree for the neurons in Fig. 2 and distribution over interactions $J_{\text{ij}}$ in the induced maximum-entropy model. The central neuron has the most connections, and those on the perimeter are leaves (with one connection) with distance from the center decreasing in the clockwise direction. (b) Same as (a) for a random tree. (c) Correlation coefficients predicted in the models vs those in the data, with the dashed line indicating equality. All pairs are divided evenly into bins along the x-axis, with solid lines and shaded regions reflecting means and standard deviations within bins. (d) Probability of the neuron to be active $x_{i}=1$ vs effective field $h_{i}^{\text{eff}}$. Solid lines are computed over all neurons and all time points, and the dashed line indicates the theoretical prediction in Eq. (11).

**FIG. 4.**
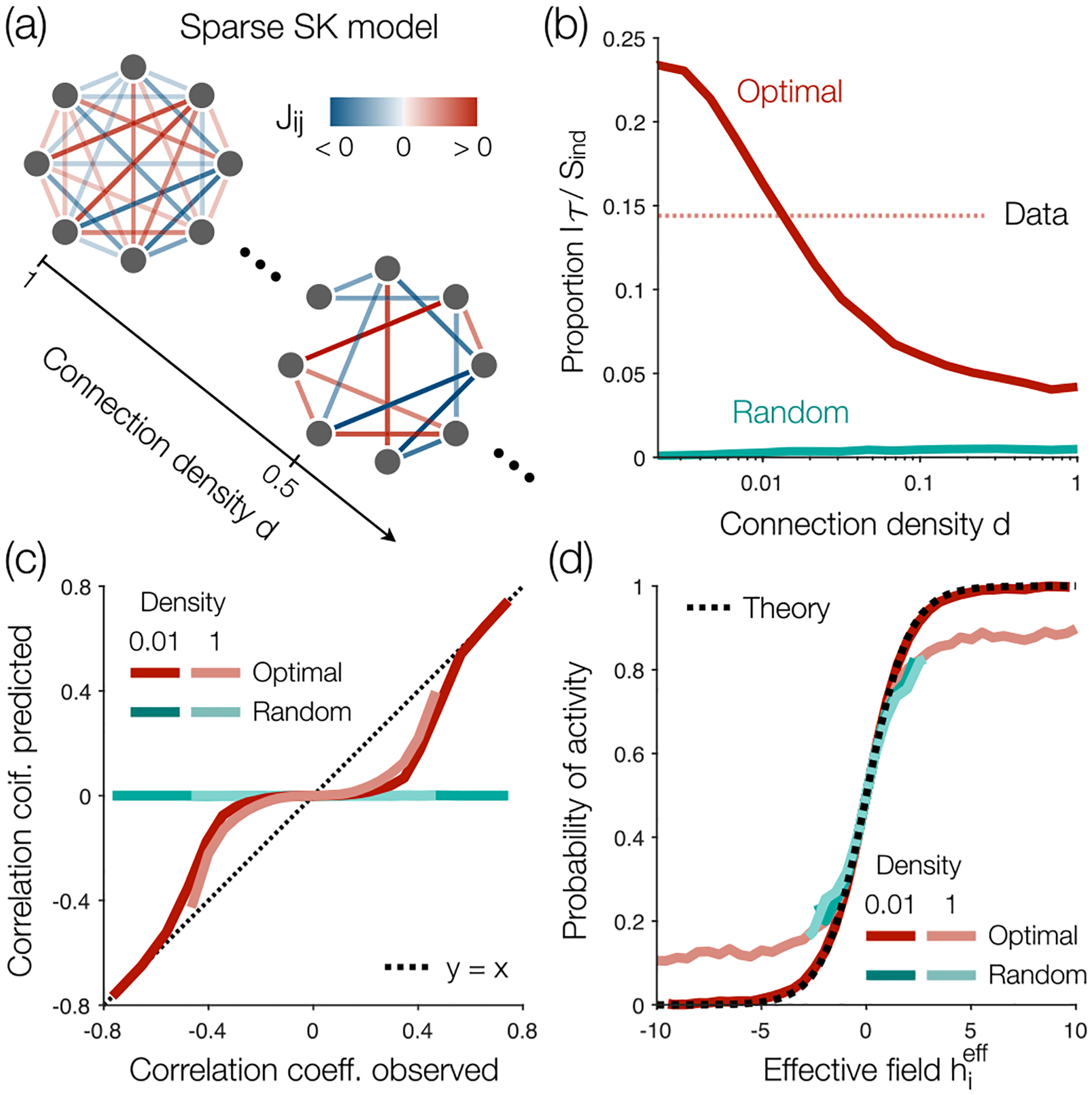
Minimax entropy model of artificial neurons. (a) Sparse SK model defined by Eq. (6) with all-to-all interactions $J_{\text{ij}}$ drawn from a zero-mean Gaussian with standard deviation $4/\sqrt{N}$ and fields $h_{i}=-\frac{1}{2}\sum_{j}J_{\text{ij}}$; this is equivalent to an Ising model with $x_{i}=\pm 1$, zero fields, and Gaussian interactions with standard deviation $1/\sqrt{N}$. We introduce sparsity by randomly removing 1-d of the interactions and rescaling the strength of interactions to maintain a constant standard deviation. (b) Fractions of independent entropy $I_{𝒯}/S_{\text{ind}}$ captured by optimal and random trees as functions of the connection density d. Each point is averaged over 10 Monte Carlo simulations of the SK model with N=1485 neurons and $M\approx 7\times 10^{4}$ samples to match the data. Dashed line indicates the value of the optimal tree in neural data $I_{𝒯}/S_{\text{ind}}\approx 14.4\%$. (c) Correlation coefficients predicted by optimal and random trees vs those observed in the SK model. (d) Probability of $x_{i}=1$ vs effective field $h_{i}^{\text{eff}}$, with the dashed line indicating the theoretical prediction in Eq. (11). In (c) and (d), each line represents a single Monte Carlo simulation.

**FIG. 5.**
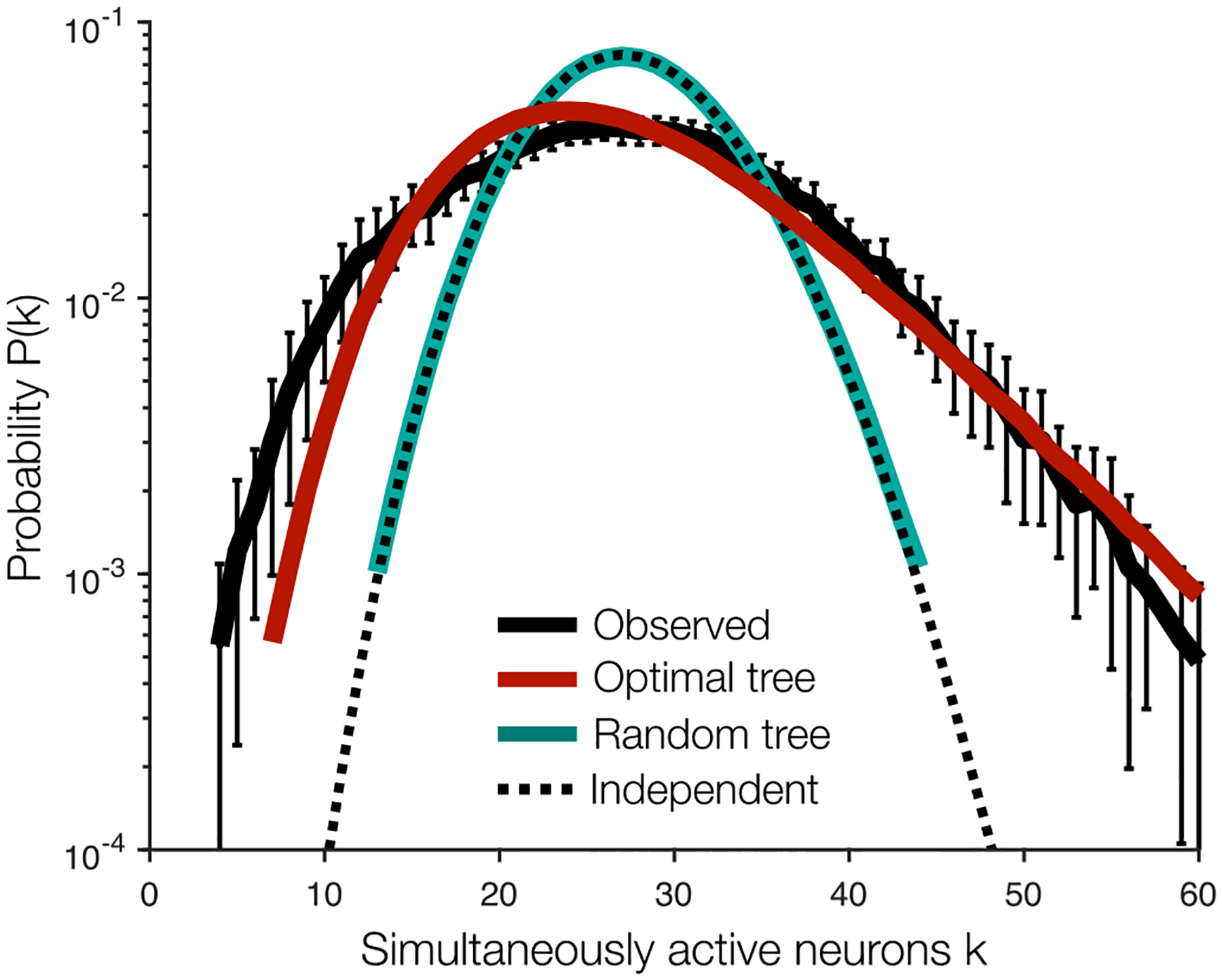
Predicting synchronous activity. Distribution P(k) of the number of simultaneously active neurons k in the data (black) and predicted by the optimal tree (red), a random tree (cyan), and independent neurons (dashed). To estimate probabilities P(k) and error bars (two standard deviations), we first split the experiment into 1-min blocks to preserve dependencies between consecutive samples. We then randomly select one-third of these blocks and repeat 100 times. For each subsample of the data, we compute model predictions for P(k) using Monte Carlo simulations with the same number of samples as the data.
